# The effect of the initial administration of suvorexant on severe sleep apnea syndrome

**DOI:** 10.1007/s41105-024-00548-7

**Published:** 2024-09-12

**Authors:** Yuki Mieno, Masamichi Hayashi, Tomohide Souma, Tomoya Horiguchi, Yoshikazu Niwa, Shiho Fujita, Jyunichi Fukumoto, Nami Hosoda, Kazuyoshi Imaizumi

**Affiliations:** 1 Department of Respiratory Medicine, School of Medicine, Fujita Health University, 1-98, Dengakugakubo, Kutsukake-cho, Toyoake, Aichi 470-1192 Japan; 2 Fujita Health University Okazaki Medical Center, Okazaki, Aichi Japan; 3 Fujita Health University Clinical Laboratory Center, Toyoake, Aichi Japan

**Keywords:** Obstructive sleep apnea, Suvorexant, Orexin receptor antagonist, Polysomnography

## Abstract

The purpose of this study was to evaluate how the first oral administration of suvorexant affects PSG results in patients with severe obstructive sleep apnea (OSA). Single-center, prospective study conducted in a nonrandomized, uncontrolled, unblinded fashion. Undiagnosed 64 patients with suspected OSA underwent first-night PSG, and 30 patients with severe OSA (Apnea Hypopnea Index [AHI] ≥ 30 events/h) underwent second-night PSG testing after administration of 15 mg suvorexant. The change in AHI between the first and second nights was not significant, although the upper limit of the 95% confidence interval for the mean difference in AHI was high at 5.987.The mean duration of apnea on the second night was significantly prolonged compared to that on the first night, but there were no significant differences n 3% oxygen desaturation index, saturation of percutaneous oxygen<90% time. On the second night, total sleep time was significantly prolonged, mid-night awakenings decreased, REM sleep percentage increased, and REM latency was shorter. Because the environment for PSG testing is very different from the patient's home and many patients have difficulty sleeping, there are clinical cases in which PSG is performed with sleep medication. In this study, PSG after oral administration of 15 mg of suvorexant on the second night showed no significant difference or clear trend in AHI. However, the upper limit of the 95% confidence interval for the mean difference in AHI was greater than 5, suggesting that suvorexant may exacerbate AHI, even with the first administration.

## Introduction

Obstructive sleep apnea (OSA) is a sleep-related breathing disorder characterized by recurrent partial or complete airway obstruction in the mid-pharynx during sleep. Intermittent hypoxemia and arousals occur during sleep. The associated increased sympathetic activity, inflammation, endothelial dysfunction, and elevated blood pressure are associated with an increased risk of coronary morbidity and mortality [1].

The prevalence of OSA among different populations varies considerably, with the prevalence of an Apnea Hypopnea Index (AHI) ≥ 5 events/h reported to be more than 30% in many cases [2]. In Japan, the prevalence of AHI ≥ 5 events/hour is reported to be 32.7% and 14.0% for AHI ≥ 15. [2] Globally, it is estimated that approximately 425 million adults aged 30–69 years have moderate OSA (AHI ≥ 15 events/h) [2]. However, many patients do not present with typical symptoms, and therefore remain undiagnosed [3] [4].

OSA and insomnia frequently coexist [5,6]. A recent meta-analysis assessing global and regional prevalence based on World Health Organization regions found that 38% of patients with OSA had concomitant insomnia [7]. Continuous positive airway pressure (CPAP), the most recommended treatment for OSA, is known to be less tolerable in patients with comorbid insomnia [8,9]. It is recommended that both OSA and insomnia be treated [10] [11].

Benzodiazepines, which are commonly used to treat insomnia, bind to the receptors in the γ-aminobutyric acid (GABA)-A complex and are associated with suppressed central respiratory drive, a blunted arousal response to hypoxia, and decreased upper airway muscle tone [12,13], and thus can exacerbate nocturnal hypoxia in patients with OSA. Conversely, nonbenzodiazepines have hypnotic and sedative effects similar to those of benzodiazepines, especially those selective for GABA receptors, including the α1 subunit, but less pronounced muscle-relaxant effects. Thus, they are considered a promising alternative for treating patients with both OSA and symptoms of insomnia. [11,14] A meta-analysis evaluating the effects of nonbenzodiazepine sleep medications on AHI found that they did not worsen AHI regardless of the severity of OSA, and in many cases slightly improved AHI compared to that in the placebo group [13,15]. However, in drug-specific evaluations, a study of OSA patients undergoing CPAP therapy found that the administration of zaleplon and zolpidem reduced the lowest levels of percutaneous oxygen saturation., it was found that the administration of zaleplon and zolpidem reduced the lowest levels of percutaneous oxygen saturation (SpO_2_) [16], whereas eszopiclone was found to reduce AHI [17], indicating that different nonbenzodiazepines can have different effects on sleep-related parameters.

In recent years, the use of orexin receptor antagonists has emerged as a novel strategy for the treatment of insomnia. Moreover, Suvorexant has been demonstrated to exert minimal impact on a patient's neurophysiology, as assessed using Electroencephalography (EEG) [18], and that, suvorexant and zaleplon have been observed to preserve the overall sleep architecture in individuals with insomnia, with the exception of a mean 3.9% increase in rapid eye movement (REM) sleep duration [16,19]. Suvorexant is the world’s first orexin receptor antagonist, released in Japan in 2014. Suvorexant is a reversible antagonist with high selectivity for human orexin 1 (OX1) and orexin 2 (OX2) receptors. By reversibly inhibiting the binding of orexins A and B, neuropeptides that promote wakefulness, to OX1 and OX2 receptors, it is thought to induce sleep by moving the brain from a waking state to a sleep state [20]. Suvorexant has little muscle relaxant effect and is considered to have a low risk of falls due to muscle relaxation during awakenings from sleep [21,22]. Suvorexant did not exacerbate SpO_2_ during sleep in healthy participants or patients with mild to moderate OSA at 40 mg (twice the maximum recommended approved dose) [23,24], and Lemborexant, another orexin receptor antagonist, did not exacerbate SpO2 or mean AHI during sleep at a dose of 10 mg [25].

However, there are no reports on the clinical effects of first administration of suvorexant on severe OSA. The purpose of this study was to evaluate the effects of orally administered suvorexant on sleep-disordered breathing in patients with severe OSA using polysomnography (PSG).

## Methods

### Participants

This single-center, prospective study was conducted at Fujita Medical University, Japan. All undiagnosed patients (aged ≥ 20 years) with suspected OSA who visited our outpatient clinic between December 2016 and February 2021 and were scheduled to undergo PSG were asked to participate in the study. We informed all patients that if their AHI was greater than 30 events/h on the first night, they would receive suvorexant on the second night. Patients with known or suspected complications such as insomnia, narcolepsy, circadian rhythm disorder, parasomnia, REM sleep behavior disorder, and idiopathic hypersomnia; patients taking sleep medication or antidepressants; pregnant women; and patients without sufficient judgment or consciousness were excluded from the study. These matters were confirmed by a physician interview during the outpatient visit.

**Fig. 1:**
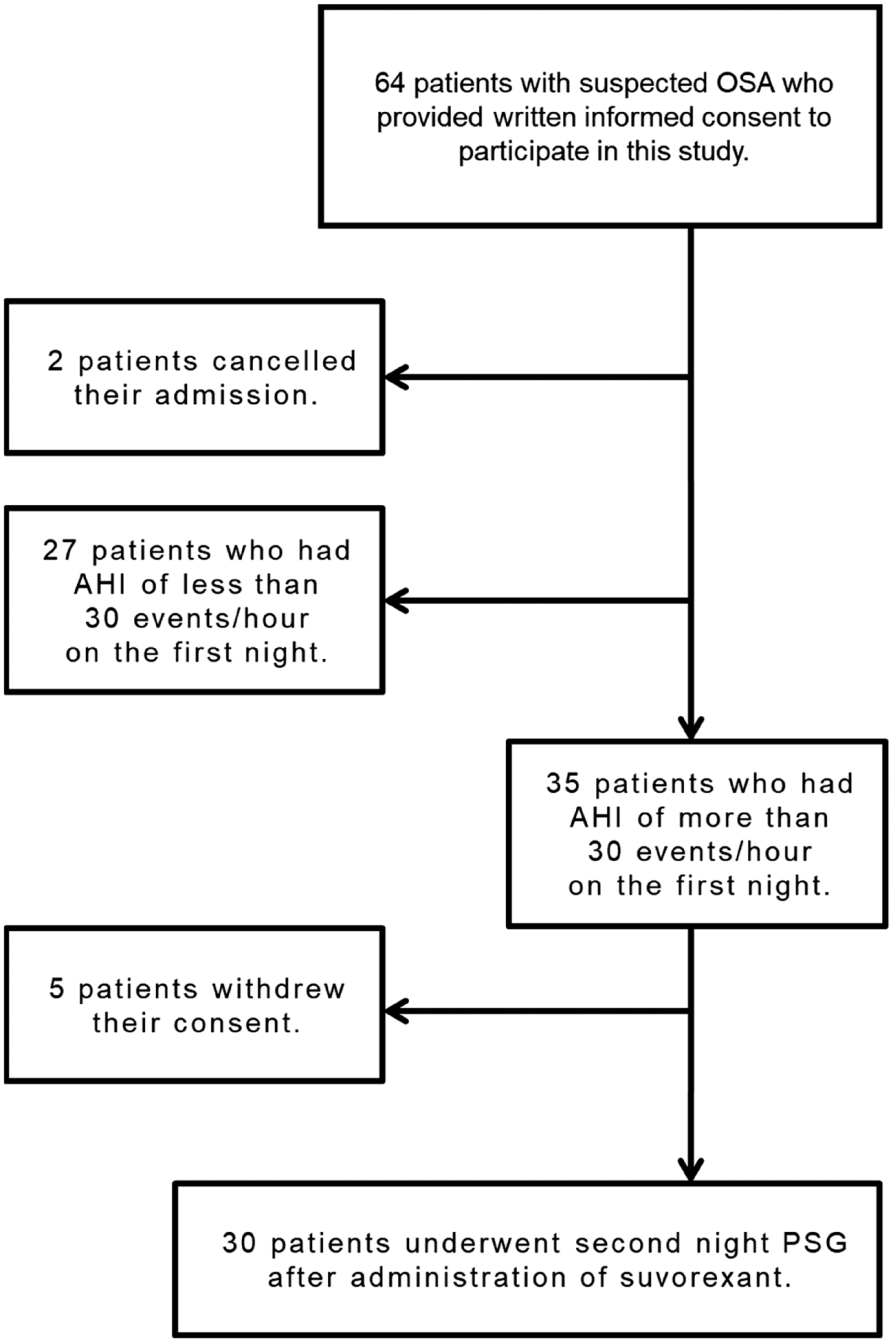
Flowchart of patient selection. Written informed consent for participation in the study was obtained from 64 patients with suspected OSA. 2 patients cancelled their admission, and 62 patients underwent first-night PSG. Of these, 35 patients had severe OSA (AHI ≥ 30 events/h). Of these, 30 patients, excluding 5 patients who withdrew consent, underwent second night PSG after administration of suvorexant

Accordingly, written informed consent for participation in the study was obtained from 64 patients with suspected OSA. Two patients cancelled their admission, and 62 patients underwent first-night PSG. Of these, 35 patients had severe OSA (AHI ≥ 30 events/h). Of these, 30 patients, excluding five who withdrew consent, underwent a second night PSG after administration of suvorexant (Fig. 1).

This study was approved by the Ethics Review Committee, Fujita Health University and Fujita Health University Certified Review Board (Approval No. HM19-059) and has been registered with the Japan Registry for Clinical Trials (“Safety Evaluation of Suvorexant, an Orexin Receptor Antagonist, for Severe Sleep Apnea Syndrome”; ID: jRCTs041180086).

### PSG

All participants were admitted to Fujita Medical University Hospital for two nights and three days, and type-1 PSG was conducted in the sleep disorder laboratory. Thirty patients with AHI ≥ 30 events/h (indicative of severe OSA) in the first-night PSG were given a single dose of suvorexant (15 mg) just before bedtime on the following night, and PSG was then performed again. On the morning of the third day, auto-CPAP was prescribed at discharge to all patients, and at-home treatment was initiated.

Twenty-eight patients underwent PSG using the SOMNOscreen system (SOMNOmedics GmbH, Germany), whereas the SomnoStar system (Vyaire Medical, USA) was used for two patients. PSG included EEG, electro-oculography, electromyography of the chin and tibialis anterior muscles, and electrocardiography. Airflow in the nasal and oral cavities was measured using heat and pressure sensors. An inductance sensor with a strain gauge was used to measure ventilatory motion of the chest and abdomen. SpO₂ was measured using a pulse oximeter. Body position measurements were recorded using accelerometers on the chest and abdominal belts.

Only SpO2 data were automatically analyzed, while other data were manually analyzed by a technician certified by the American Sleep Society as a Registered Polysomnographic Technologist. AHI was calculated by dividing the sum of apnea and hypopnea events by total sleep time and expressed in terms of the average number of events per hour. Apnea was defined as the signal amplitude of the thermal sensor in the lower nasal cavity decreasing by ≥ 90% for ≥ 10 s. Hypopnea was defined as the signal amplitude of the pressure sensor in the same area decreasing by ≥ 30% for ≥ 10 s, and SpO₂ decreasing by ≥ 3%, or when an arousal response was observed. The definitions of apnea, hypopnea, and sleep stages were in accordance with the Manual for Determining Sleep and Associated Events ver. 2.5 published by the American Academy of Sleep Medicine [26].

### Statistical analysis

Power calculations were performed for 80% power, assuming a one side significance level of 0.025, and a non-inferiority margin of 5 for the difference in AHI between the first and second nights.

As the primary endpoint, the change between the AHI on the first and second nights was compared using the paired *t*-test. To evaluate the effects on respiration and oxygenation, the apnea index, 3% oxygen desaturation index, mean apnea duration, SpO₂ < 90% time, and lowest SpO₂ on the first and second nights were compared as secondary endpoints using the paired *t*-test. Total sleep time, sleep efficiency, mid-night awakenings, sleep stages duration percentages, arousal index, sleep latency, and REM latency were also compared to evaluate the impact on sleep.

Finally, linear regression analysis was used to examine the effects of age and body mass index (BMI), with the difference between the values of each parameter on the first and second nights as the independent variables and age and BMI as dependent variables.

The Japanese version of JMP 14 (SAS Institute Inc., Tokyo) was used for all statistical analyses, and the significance level was set at *p* < 0.05.

## Results

### Patient characteristics

All 30 participants with severe OSA (25 men and five women) completed two consecutive nights of PSG testing. The median age and median BMI were 55 (interquartile range [IQR], 50.8–60.8) years and 26.3 (IQR, 24.8–29.0) kg/m^2^, respectively. Median Epworth Sleepiness scale score and median AHI on the first night were 8.0 (IQR, 3.8–11.3) and 41.6 (IQR, 37.1–61.4) events/h, respectively (Table 1).

**Table 1:** Patient characteristics (*n* = 57)

		Group of AHI < 30/h (*n* = 27)	Group of AHI≧30/h (*n* = 30)	*P*-value
Gender	%(male)	81.5	83.3	0.6833
Age	years old	54 (45.0–63.0)	55 (50.8–60.8)	0.8544
Body mass index	kg/m^2^	22.9 (21.7–25.5)	26.3 (24.8–29.0)	< 0.0001
Epworth sleepiness scale		10.0 (6.0–11.0)	8.0 (3.8–11.3)	0.1566
First night AHI	Events/hour	14.4 (10.3–22.5)	41.6 (37.1–61.4)	< 0.0001

Data, with the exception of gender, are presented as median (interquartile range). Gender was compared using the χ2 test, and all other data were compared using Wilcoxon's test

### Primary endpoint

**Fig. 2:**
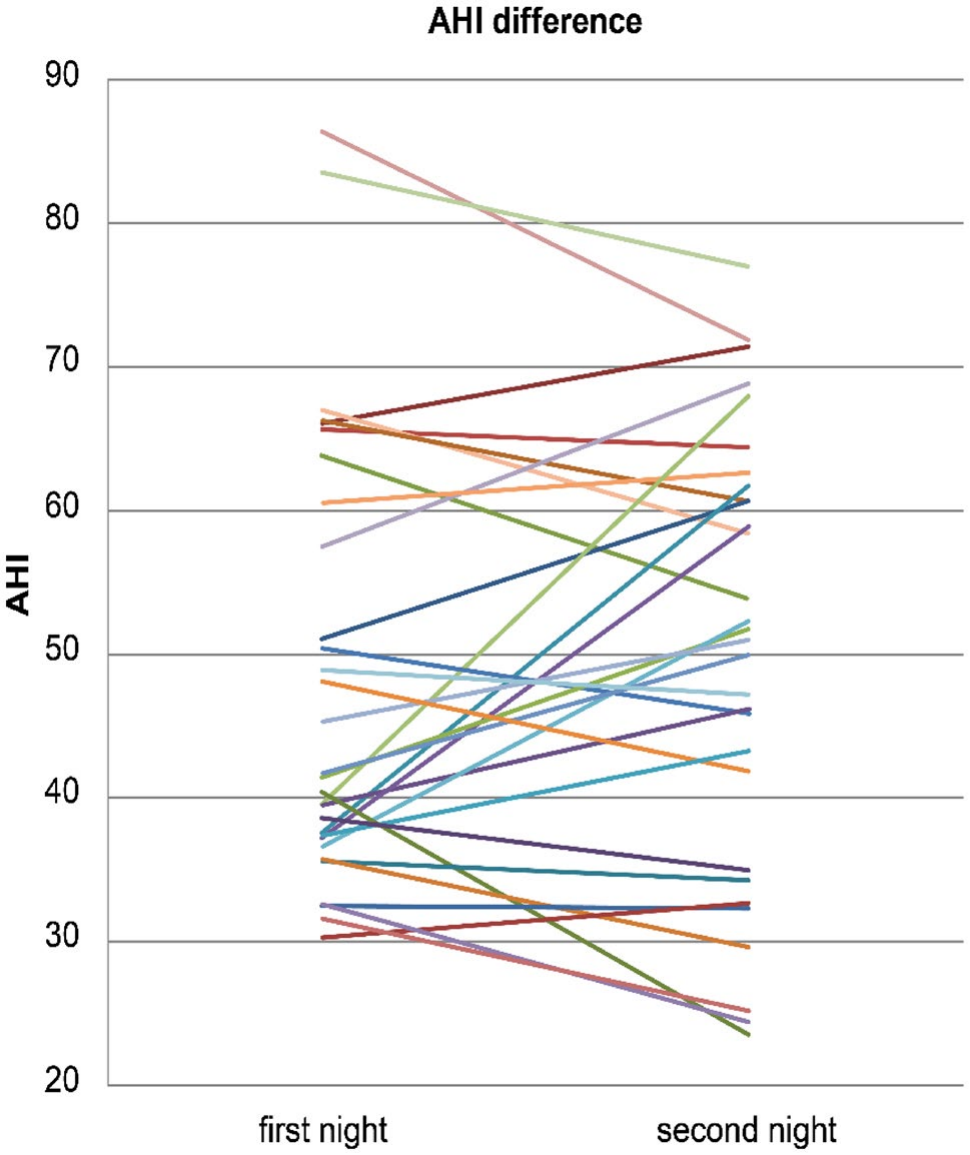
Comparison of AHI between the first and second night. Mean of difference 1.873, 95% confidence interval (− 2.241 to 5.987), *p* = 0.3594

The change in AHI between the first and second nights was not significant, but the upper 95% confidence interval (CI) limit was greater than 5 (mean difference: 1.87, 95% CI: [− 2.24, 5.98], *p* = 0.3594) **(**Fig. 2**)**.

Twenty-two (73%) of the 30 patients with severe OSA had an AHI change of at least 5 events/h (increase in 12 cases, decrease in 10 cases) between the first and second night, whereas eight patients had an AHI change of at least 10 events/h (increase in six cases, decrease in two cases). On the second night, four patients (13%) had an AHI of less than 30 events/h, indicating a change from severe to moderate disease.

### Secondary endpoints

Effect on respiration: The mean duration of apnea on the second night was significantly prolonged compared to that on the first night (mean difference: 1.97 s, 95% CI: [0.20, 3.74], *p* = 0.031). There were no significant differences in apnea index, 3% oxygen desaturation index, SpO₂ < 90% time, or lowest SpO₂ during sleep between the first and second nights (Table 2).

**Table 2:**
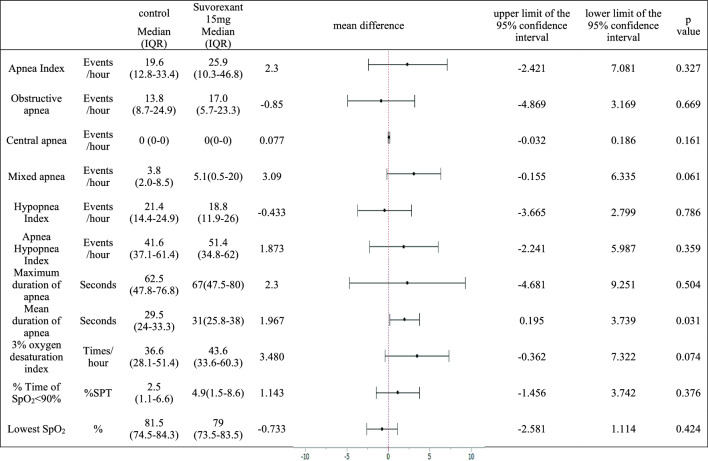
Effects of Suvorexant on Sleep-Related Parameters on PSG

Comparison of PSG measurements between the first and second night using the T-test with correspondence

Effect on sleep: Total sleep time was significantly prolonged on the second night compared to that on the first night (mean difference: 15.37 min, 95% CI: [2.96, 27.73], *p* = 0.017). Furthermore, sleep efficiency increased (mean difference: 5.04, 95% CI: [1.76, 8.32], *p* = 0.024), mid-night awakenings decreased (mean difference: − 3.347, 95% CI: [− 5.92, − 0.78], *p* = 0.013), REM sleep percentage increased (mean difference: 6.34, 95% CI: [4.14, 8.53], *p* < 0.0001), and REM latency was shorter (mean difference: − 50.2 min, 95% CI: [− 79.70, − 20.71], *p* = 0.002) on the second night. There were no significant differences in the percentages of any non-REM sleep stage (N1, N2, or N3), arousal index, or sleep latency (Table 3).

**Table 3:**
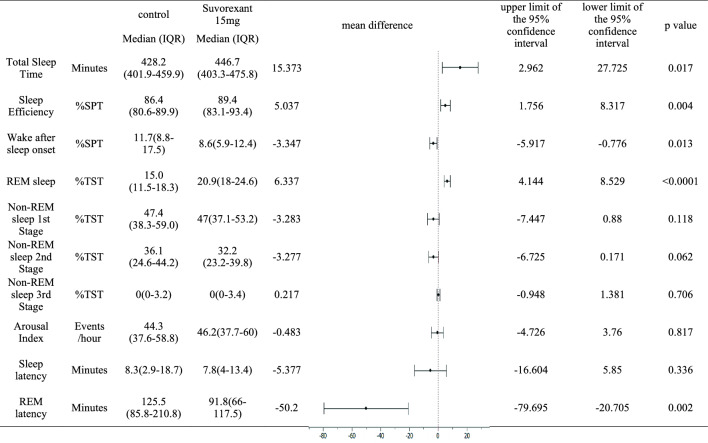
Effect of Suvorexant on Respiration-Related Parameters on PSG

Comparison of PSG measurements between the first and second night using the T-test with correspondence

The results of the linear regression analysis showed that age and BMI were not significantly correlated with the changes in any respiration- or sleep-related parameters (Table 4).

**Table 4:** Effect of age and BMI on the difference between each measurement on PSG

		Estimated values	Lower limit of the 95% confidence interval	Upper limit of the 95% confidence interval	p value
AHI difference	Age	0.034	− 0.360	0.426	0.859
	BMI	0.770	− 0.212	1.754	0.119
Apnea Index difference	Age	− 0.023	− 0.488	0.442	0.922
	BMI	− 0.523	− 1.691	0.644	0.366
Mean duration of apnea difference	Age	− 0.007	− 0.185	0.171	0.936
	BMI	0.002	− 0.444	0.449	0.991
3% oxygen desaturation index difference	Age	0.102	− 0.271	0.475	0.579
	BMI	− 0.365	− 1.302	0.572	0.431
% Time of SpO_2_ < 90% difference	Age	− 0.008	− 0.268	0.252	0.951
	BMI	0.089	− 0.564	0.742	0.781
Lowest SpO_2_ difference	age	0.006	− 0.179	0.191	0.947
	BMI	0.080	− 0.384	0.544	0.727
Total Sleep Time difference	Age	0.876	− 0.310	2.061	0.141
	BMI	0.307	− 2.669	3.284	0.834
Sleep Efficiency difference	Age	0.046	− 0.264	0.356	0.763
	BMI	− 0.550	− 1.329	0.228	0.159
Wake after sleep onset difference	Age	− 0.062	− 0.317	0.192	0.619
	BMI	0.111	− 0.528	0.750	0.725
REM sleep difference	Age	0.001	− 0.219	0.221	0.991
	BMI	0.027	− 0.525	0.579	0.921
Non-REM sleep 1st Stage difference	Age	0.061	− 0.343	0.46	0.759
	BMI	− 0.510	− 1.523	0.50	0.311
Non-REM sleep 2nd Stage difference	Age	− 0.012	− 0.345	0.320	0.940
	BMI	0.515	− 0.319	1.350	0.216
Non-REM sleep 3rd Stage difference	Age	− 0.050	− 0.165	0.006	0.378
	BMI	− 0.030	− 0.319	0.259	0.833
Arousal Index difference	Age	0.065	− 0.332	0.46	0.738
	BMI	− 0.769	− 0.332	0.46	0.125
Sleep latency difference	Age	0.791	− 0.287	1.87	0.144
	BMI	0.384	− 1.325	4.09	0.304
REM latency difference	Age	0.4	− 2.536	3.321	0.785
	BMI	2.644	− 1.708	9.997	0.467

A linear regression analysis was performed with the difference between the first and second night of each measurement as the objective variable and age and BMI as dependent variables

## Discussion

The results of this study show that oral administration of 15 mg of suvorexant did not result in significant changes in AHI in patients with severe OSA. To the best of our knowledge, this is the first study to assess the effects of an orexin receptor antagonist on patients with severe OSA.

The recommended doses of suvorexant in the United States and Japan are 15 mg for elderly patients and 20 mg for adults. A previous study that examined the effects of a single dose of 40 mg of suvorexant on patients with mild to moderate OSA found no significant difference in mean SpO_2_ during the total sleep time, and concluded that 40 mg of suvorexant had no clinically significant sleep-disordered breathing effects on patients with mild to moderate OSA [24]. However, because the safety of suvorexant in patients with severe OSA has not been confirmed, in this study, we examined the use of the lowest clinically used dose of 15 mg in Japan.

We did not observe a significant difference between AHI on the first night and that after oral administration of 15 mg suvorexant on the second night (mean difference: 1.873, 95% CI: [− 2.241, 5.987], *p* = 0.359). Power calculations were performed with reference to a previous study [27]; accordingly, it was calculated that a minimum of 24 cases would be required and that therefore 30 cases would be sufficient for this study. The AHI is widely used for the diagnosis and severity classification of OSA [28] and has been used in many studies examining respiratory safety in patients with OSA [24,28]. As it has been noted that an increase in AHI of 5 events/h increases the risk of hypertension and type 2 diabetes [17,29],this difference is generally used as a clinically meaningful criterion in studies on the safety of sleep medication for patients with OSA [23,24,27]. Therefore, the non-inferiority margin was set at 5 in this study as well, but the upper limit of the 95% CI was found to be 5.98, which is greater than 5, and therefore non-inferiority could not be proven. Compared to the latest study [27] that examined the effect of orexin receptor antagonists on OSA, the standard deviation of AHI in the population of this study was 15.3, which was larger than that of AHI of 8.49 in the population of the previous study [27]. This may have contributed to the larger 95% CI.

Of the 30 patients with severe OSA, 22 (73%) had an AHI change of 5 events/h or more (increase in 12 cases, decrease in 10 cases), and eight (26%) had an AHI change of 10 events/h or more (increase in six cases, decrease in two cases). It is known that there is night-to-night variability in PSG results [30,31]. In particular, the AHI of patients with OSA has been reported to be highly variable across multiple PSG trials [31,32]. Since the AHI varied around 10 events/h in 32–49% of patients with OSA during two consecutive nights of PSG [33,34], the AHI change in this study may have been due to the night-to-night variability that can occur in PSG.

The 3% oxygen desaturation index and SpO₂ < 90% time are also important indicators of respiratory safety [28,35]. In the present study, the mean duration of apnea was significantly prolonged (mean difference: 1.967 s, 95% CI: [0.195, 3.74], *p* = 0.031). However, all the parameters related to oxygenation, such as the 3% oxygen desaturation index, SpO₂ < 90% time, and lowest SpO₂, showed no significant difference between the first and second nights, although the CI for the 3% oxygen desaturation index tended to be slightly higher (Table 2).

First-night effects on sleep have been reported to decrease total sleep time, increase mid-night awakenings, and disrupt sleep structure (increase N1 sleep, decrease REM sleep, and prolong REM latency) [31]. In this study, total sleep time was significantly prolonged, sleep efficiency increased, and mid-night awakenings were reduced on the second night compared to those on the first night. These results are consistent with the release of the first-night effect. However, although there was no significant difference in the percentage of N1, N2, and N3 sleep stages between the two nights, the increase in the percentage of REM sleep and the shortening of REM latency were thought to be influenced by suvorexant as well as the first-night effect. A previous study on suvorexant reported that total sleep time was prolonged and REM sleep increased after the first night of administration, but that there was no significant difference in the percentage of non-REM sleep [24], which is consistent with this study.

It is known that some OSA patients exhibit an increase in AHI during REM sleep (REM AHI is more than twice the NREM AHI) [36]. In this study, four patients were identified as having REM-dependent OSA on the first night of PSG. Among these four patients, three did not exhibit REM dependency on the second night, and no new patients exhibited REM dependency on the second night.

A paired t-test comparing the AHI on the first and second nights for these four patients showed no significant difference (mean difference: 11.2, 95% CI: [− 16.8, 39.25], *p* = 0.2922). Similarly, a paired *t*-test comparing REM-AHI between the two nights also showed no significant difference (mean difference: − 9.78, 95% CI: [− 25.93, 6.38], *p* = 0.1497). Therefore, our study did not find clear evidence that suvorexant administration increases AHI in patients with REM-dependent OSA. However, it is important to note that the sample size for REM-dependent cases was small, with only four patients on the first night. This may not be sufficient for statistical analysis.

Additionally, we examined the correlation between changes in AHI and changes in the percentage of REM sleep over the two nights. The correlation coefficient was − 0.172, with a 95% confidence interval of − 0.52 to 0.201 and a *p*-value of 0.363, indicating no significant correlation.

Linear regression analysis using the differences between values of each PSG parameter on the first and second nights as the objective variables and age and BMI as dependent variables showed no significant effect of age or BMI on either respiration-related or sleep-related parameters. Thus, age and BMI did not appear to have a significant effect on any of the parameters in this study population.

In summary, while this study found no significant changes in AHI and other respiration-related parameters while demonstrating some effects of suvorexant, such as increased REM sleep percentage and shortened REM latency, the upper limit of the 95% CI for the difference in AHI was 5.98, which is slightly more than 5, which did not prove non-inferiority. The changes in the other parameters can be interpreted as due to night-to-night variability.

Some limitations of this study must be noted. As this study was based on PSG testing on two different nights, night-to-night variability cannot be ruled out.　Moreover, as a crossover study is desirable when examining safety, the results should be interpreted with caution. The presence of confounding factors other than age and BMI also cannot be ruled out. Additionally, the study participants and technicians who assessed PSG were not blinded, due to which participant and observer bias could not be eliminated, which limits the external validity of the study.

## Conclusions

Because the environment for PSG testing is very different from the patient's home and many patients have difficulty sleeping, there are clinical cases in which PSG is performed with sleep medication. This is the first study to examine the effects of first administration of the orexin receptor antagonist suvorexant on patients with severe OSA. PSG after oral administration of 15 mg suvorexant on the second night showed an increase in the percentage of REM sleep and a shortening of REM latency, but no significant difference or clear trend regarding AHI, although this could have been due to night-to-night variability. However, the upper limit of the 95% confidence interval for the mean difference in AHI was greater than 5, suggesting that suvorexant may exacerbate AHI, even with the first administration. Future studies should be conducted with sample sizes and study designs that assume a more realistic standard deviation of the AHI.

## Declarations

### Research involving human and animal participants

All procedures performed in studies involving human participants were in accordance with the ethical standards of the institutional and/or national research committee and with the 1964 Helsinki Declaration and its later amendments or comparable ethical standards. This article does not contain any studies with animals performed by any of the authors.
